# Changes in lactate dehydrogenase on admission throughout the COVID-19 pandemic and possible impacts on prognostic capability

**DOI:** 10.2217/bmm-2022-0364

**Published:** 2022-09-02

**Authors:** Elba O Medina-Hernández, Lucía M Pérez-Navarro, Joselín Hernández-Ruiz, Alma Villalobos-Osnaya, María L Hernández-Medel, Catalina Casillas-Suárez, Adolfo Pérez-García

**Affiliations:** ^1^Nephrology Service, Hospital General de México ‘Dr Eduardo Liceaga’, Mexico; ^2^Research Department, Hospital General de México ‘Dr Eduardo Liceaga’, Mexico; ^3^Nephrology Department, School of Medicine, University of Utah, UT, USA; ^4^Infectology Service, Hospital General de México ‘Dr Eduardo Liceaga’, Mexico; ^5^Pneumology Service, Hospital General de México ‘Dr Eduardo Liceaga’, Mexico

**Keywords:** area under the curve, COVID-19, LDH, prognostics, relative risk, sensitivity, specificity, triage

## Abstract

**Introduction:** The enzyme lactate dehydrogenase (LDH) is a good marker of general hyperinflammation correlated with mortality for COVID-19, and is therefore used in prognosis tools. In a current COVID-19 clinical randomized trial (CRT), the blood level of LDH was selected as an inclusion criterion. However, LDH decreased during the pandemic; hence, the impact of this decrease on the prognostic value of LDH for mortality was evaluated. **Methods:** Data on LDH levels in 843 patients were obtained and analyzed. Relative risk, standard error and receiver operating characteristic curves were calculated for two cutoff values. **Results:** Relative risk lost validity and the area under the curve narrowed by trimester during the pandemic. **Conclusion:** The progressive decrease in LDH impacted the capacity to predict mortality in COVID-19. More studies are needed to validate this finding and its implications.

The need for a quick, reliable and low-cost prognostic tool for COVID-19 has elicited multiple studies focused on finding the best prognostic variables to estimate outcomes, some based on clinical characteristics, some on biomarkers and some on both, either alone or combined with other existing tools, such as the Sequential Organ Failure Assessment [1–3]. In Mexico, three scores have been developed: the Bello-Chavolla and patient history (PH)-COVID-19 scores based on age and risk factors [4,5], and the low-harm score [6], which takes into account other factors, such as oxygen saturation and leukocytes.

In COVID-19, severe and critical phases are caused by an exacerbated dysfunctional immune response that leads to a life-threatening condition known as cytokine release syndrome or ‘cytokine storm’ [7,8]. Therefore, prognostics are oriented toward identifying key indicators of the onset and development of hyperinflammatory states. The glycolytic enzyme lactate dehydrogenase (LDH) has long been identified as a systemic inflammation biomarker and is therefore considered a good prognostic indicator under general inflammatory conditions [9,10]. LDH belongs to the oxidoreductase class and has an important role in the anaerobic glycolysis pathway, thus being present in practically all human cells [11]. It is a stable cytoplasmic enzyme that increases in the bloodstream under conditions of membrane instability [12]. LDH is commonly used in clinical practice as a marker of cardiac damage/necrosis [13]. Moreover, some studies have identified LDH as an especially useful marker for the evaluation and prognosis of inflammation-related pulmonary conditions [14,15]. Furthermore, LDH quantification is a routine, low-cost and accessible process.

One therapeutic strategy for COVID-19 is designed to achieve an immunomodulatory effect that could prevent hazardous immune dysregulation. Consequently, multiple ongoing studies are evaluating the performance of some drugs to accomplish this goal by different therapeutic targets, namely CCR5 [16], IL-1 [17], IL-6 [18], IL-17 [19] and TNF-α [20]. The current authors' group at the Hospital General de Mexico ‘Dr Eduardo Liceaga’ is currently working on a protocol designed to evaluate the effect of a CCR5-targeted immunomodulator (maraviroc) and an antiviral (favipiravir), either alone or combined, to prevent the progression of severe patients to critical condition or death, compared with the standard treatment [21]. Serum LDH level was selected as an inclusion criterion since it was expected to be a good indicator in discriminating those patients prone to require mechanical ventilation or die in the first 48 h postadmission. Therefore, LDH levels for 330 PCR-confirmed COVID-19 patients admitted between 9 April and 3 October 2020, were reviewed and found to average 427.66 IU/l. Taking into consideration that these data were observed during the first stage of the pandemic, in which very high mortality was observed (36%), a cutoff value of 350 IU/l was initially set for blood LDH upon admission. The first patient was recruited on 10 July 2021, during the third pandemic wave in Mexico [22]. After a brief discussion with the physicians in charge of hospital admissions, 250 IU/l was ultimately selected as the cutoff value, in order to recruit sufficient patients. However, after a relatively quick start in the first 2 weeks, recruitment progressively slowed, coming to a halt on 13 September 2021, after only 11 patients had been included. This is partly explained by the reduction in cases related to the passing of the third wave; however, this data was nevertheless notable since many patients were being evaluated as candidates. Thus, an analysis of the causes of noninclusion was undertaken using data from the first day of the study to 23 November 2021. An LDH level below the chosen cutoff was the second cause of noninclusion (44/182, 24.2%). This finding suggests that LDH levels could have decreased throughout the pandemic. Therefore, the purpose of this work was to determine whether decreases in blood LDH levels occurred among confirmed COVID-19 patients at admission, during the first 20 months of the pandemic, and if so, to evaluate the impact of these decreases on predictive capabilities for mortality. To our knowledge, this is the first retrospective cohort study to evaluate LDH as a marker of prognosis, including the largest number of patients over an extended period.

## Methods

**Figure 1.**
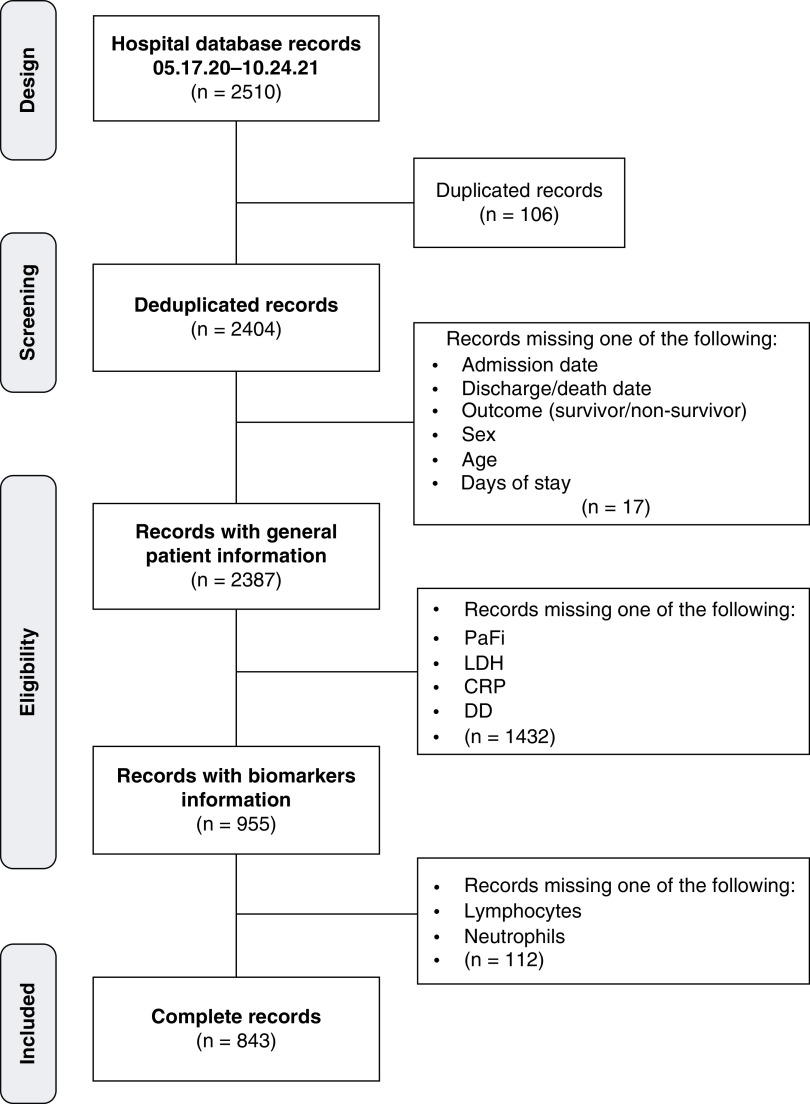
CONSORT flow diagram of the included subjects. CRP: C-Reactive protein; DD: D dimer; LDH: Lactate dehydrogenase; PaFi: Oxygen arterial saturation/Inspired oxygen fraction ratio.

This retrospective cohort study was approved by the Ethics Committee of the Hospital General de Mexico ‘Dr Eduardo Liceaga’, reg. no. DMC-3369-20-20-1. The hospital is a third-level national reference hospital in which more than 15,000 COVID patients have been treated [23]. A total of 2510 PCR-confirmed cases in adults admitted to the hospital between 17 March 2020, and 24 October 2021, were initially evaluated for data integrity. After verification, 843 cases were validated as complete for admission date, discharge/death date, outcome, sex, age, blood pressure of oxygen/inspired fraction of oxygen (PaO_2_/FiO_2_; PaFi), LDH, C-reactive protein (CRP), D-dimer (DD), lymphocytes and neutrophils (Figure 1).

Patients were first grouped by clinical outcome (survivors vs nonsurvivors). Clinical parameters on admission and length of hospital stay (days) were assessed using independent *t*-tests for age, PaFi, LDH, CRP, DD, lymphocytes and neutrophils, the median for the length of stay and the chi-square test for sex (proportion of females). Participants were then grouped by trimester according to the date of admission and compared in terms of age, sex, length of stay, mortality and LDH (the last trimester comprised only 2 months due to the origin of the data). Length of stay was compared by median test, whereas age, mortality and LDH were compared using one-way ANOVA and Scheffé *post hoc* tests (LDH underwent a logarithmical transformation for normalization for comparison purposes). A false discovery rate procedure (Benjamini-Hochberg) was performed for LDH comparisons to minimize the effect of variable group sizes. To better assess this effect, the achieved power was calculated *a posteriori*. A receiver operator characteristic (ROC) curve to determine the area under the curve (AUC), sensitivity and specificity and Youden index were calculated to determine the capacity of LDH to predict mortality in each trimester. Two cutoff values were selected (Trimesters 2 and 7). Relative risk (RR) was calculated by trimester using a binary logistic regression with a 95% CI and a power (1-β) of 80%. The significance level was set at p < 0.05 for all statistical tests. SPSS v.25, Microsoft Excel 365 MSO v. 2203, GPower v.3.1.9.7 and Epidat v3.1 were used for statistical analyses.

## Results

The results of preliminary analyses by clinical outcome and shown in Table 1. Most variables examined were similar to those reported in previous studies [24,25]. Except for the length of stay, all analyzed variables showed one or more statistical differences (p < 0.05) between trimesters.

**Table 1.** Preliminary analyses by clinical outcome.

Parameters	Total (n = 843)	Survivors (n = 377)	Deceased (n = 466)	p-value
^‡^Sex (female n, %)	292 (34.6)	139 (36.87)	153 (32.83)	<0.0001
Age (years)	56.65 ± 14.82	51.25 ± 14.32	61.01 ± 13.76	<0.0001
Length of stay (days)^†^	10 (10)	10 (9)	10 (11)	0.0623
PaFi	262.16 ± 125.80	274.96 ± 126.82	251.81 ± 124.15	0.008
Serum LDH IU/l	483.09 ± 358.11	394.87 ± 222.30	554.46 ± 425.25	<0.0001
CRP IU/l	175.41 ± 123.38	141.16 ± 115.21	203.11 ± 122.93	<0.0001
DD IU/l	4772.15 ± 13,931.63	2654.82 ± 6248.52	6485.09 ± 17,700.37	<0.0001
Lymphocytes/ml*10^3^	0.987 ± 1.22	1.16 ± 1.66	0.847 ± 0.66	<0.0001
Neutrophils/ml*10^3^	9.50 ± 5.57	7.95 ± 4.46	10.75 ± 6.04	<0.0001

Figures represent means ± standard deviation except where otherwise indicated; means compared by *t*-test except §.

† Medians (IQR) compared by median test.

‡ Chi-square test.

CRP: C-reactive protein; DD: D-dimer; IQR: Interquartile range; LDH: Lactate dehydrogenase; PaFi: Blood pressure of oxygen/inspired fraction of oxygen.

Next, participants were grouped by trimester to evaluate the behavior of the variables of interest during the pandemic. Table 2 summarizes the results of comparisons grouped by trimester. Again, length of stay did not differ between trimesters.

**Table 2.** Variables compared by trimester.

Parameters	Trimester							
	All	Mar 20–May 20, n = 228 (A)	Jun 20–Aug 20, n = 113 (B)	Sep 20–Nov 20, n = 138 (C)	Dec 20–Feb 21, n = 188 (D)	Mar 21–May 21, n = 80 (E)	Jun 21–Aug 21, n = 47 (F)	Sep 21–Oct 21, n = 49 (G)
Age (years)^†^^,^^§^	56.65 ± 14.28	53.89 ± 14.17	56.37 ± 15.22 A F	62.25 ± 13.92 A B E F G	59.37 ± 12.52 A F G	56.69 ± 15.94 A F	50.15 ± 16.62	54.69 ± 17.01
Sex ratio (female n, %)^¶^	292 (35) p < 0.0001	64 (28) p < 0.0001	38 (34) p = 0.001	46 (33) p < 0.0001	66 (61) p < 0.0001	39 (49) p = 0.82	19 (40) p = 0.18	20 (41) p = 0.19
Length of stay (days)^‡^	10 (10)	9 (10)	8 (11)	10 (11)	11 (10)	8 (9)	13 (14)	11 (14)
Mortality (deceased n, %)^§^	466 (55)	83 (36) B C D	84 (74) A E F G	116 (84) A D E F G	124 (66) A C E F G	33 (41) B C D	14 (30) B C D	12 (24) B C D

† Mean ± standard deviation.

‡ Median (IQR), median test.

§ One-way ANOVA with Scheffé *post hoc* tests.

¶ Chi-square test.

ANOVA: Analysis of variance; IQR: Interquartile range.

**Figure 2.**
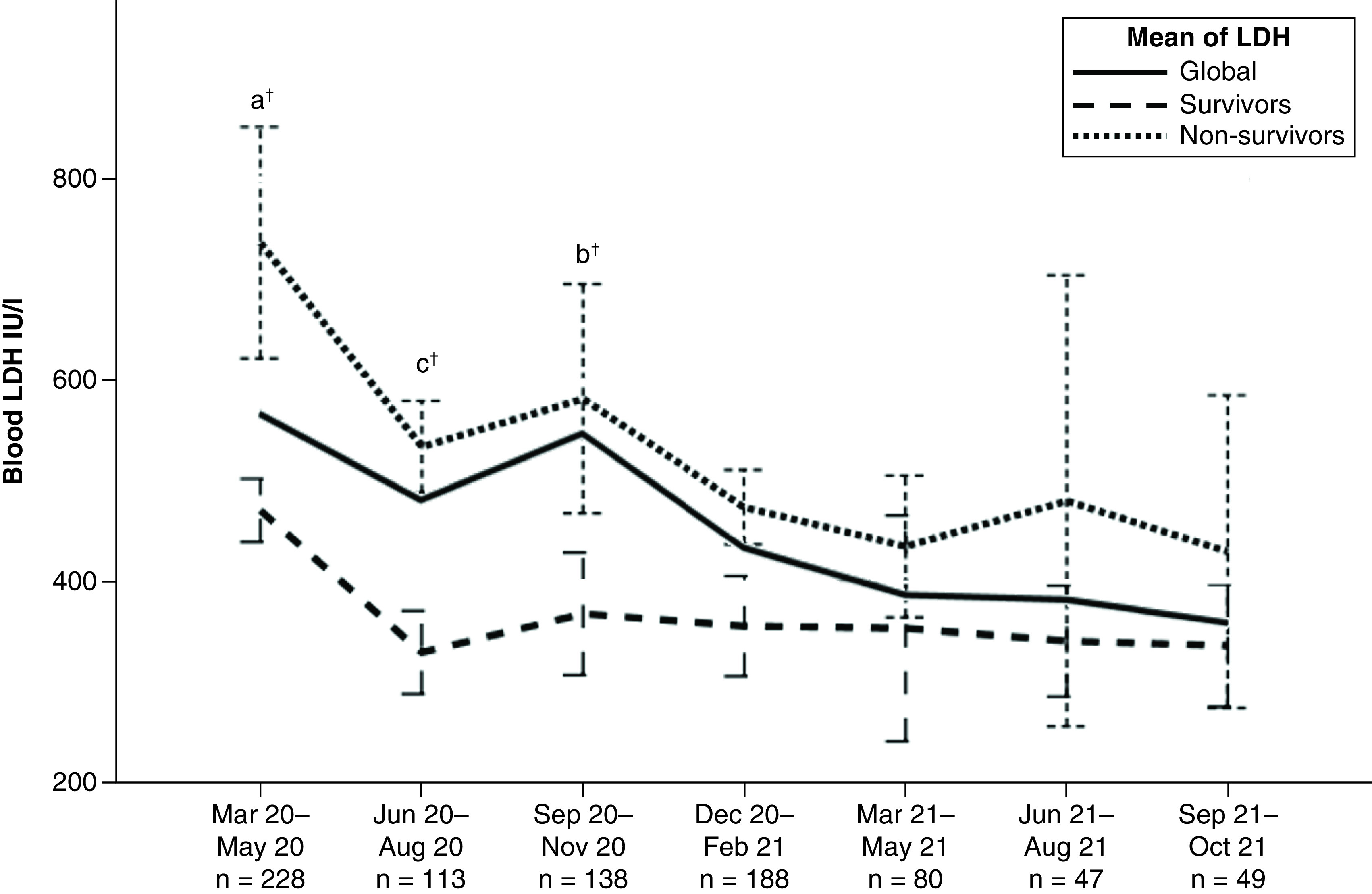
Lactate dehydrogenase at admission per trimester. ^†^p-value <0.05. LDH: Lactate dehydrogenase.

Mean LDH was compared between trimesters for all participants and survivors versus nonsurvivors (Figure 2)

A ROC curve was used to identify the highest cutoff value for LDH to predict mortality in each trimester. This cutoff value decreased as the pandemic evolved, thus behaving more like a screening test. The cutoff values by trimester are shown in Table 3.

**Table 3.** Cutoff values by trimester.

Trimester	LDH cutoff value for mortality IU/l	Sensitivity	Specificity	AUC	95% CI	p-value	Youden index
Total, n = 843	326.5	80	44	0.689	0.654–0.725	<0.0001	0.24
Mar. 20–May 20, n = 228	508	80.7	67	0.765	0.689–0.823	<0.0001	0.477
Jun. 20–Aug. 20, n = 113	340	81	59	0.81	0.728–0.897	<0.0001	0.4
Sep. 20–Nov. 20, n = 138	319.5	80	41	0.69	0.584–0.807	0.004	0.21
Dec. 20–Feb. 21, n = 188	313	80	49	0.71	0.634–0.791	<0.0001	0.29
Mar. 21–May 21, n = 80	268	81	47	0.72	0.602–0.839	0.001	0.28
Jun. 21–Aug. 21, n = 47	259	85	43	0.64	0.464–0.819	0.128	0.28
Sep. 21–Oct. 21, n = 49	243	83	30	0.65	0.45–0.85	0.109	0.13

AUC: Area under the curve; LDH: Lactate dehydrogenase.

**Figure 3.**
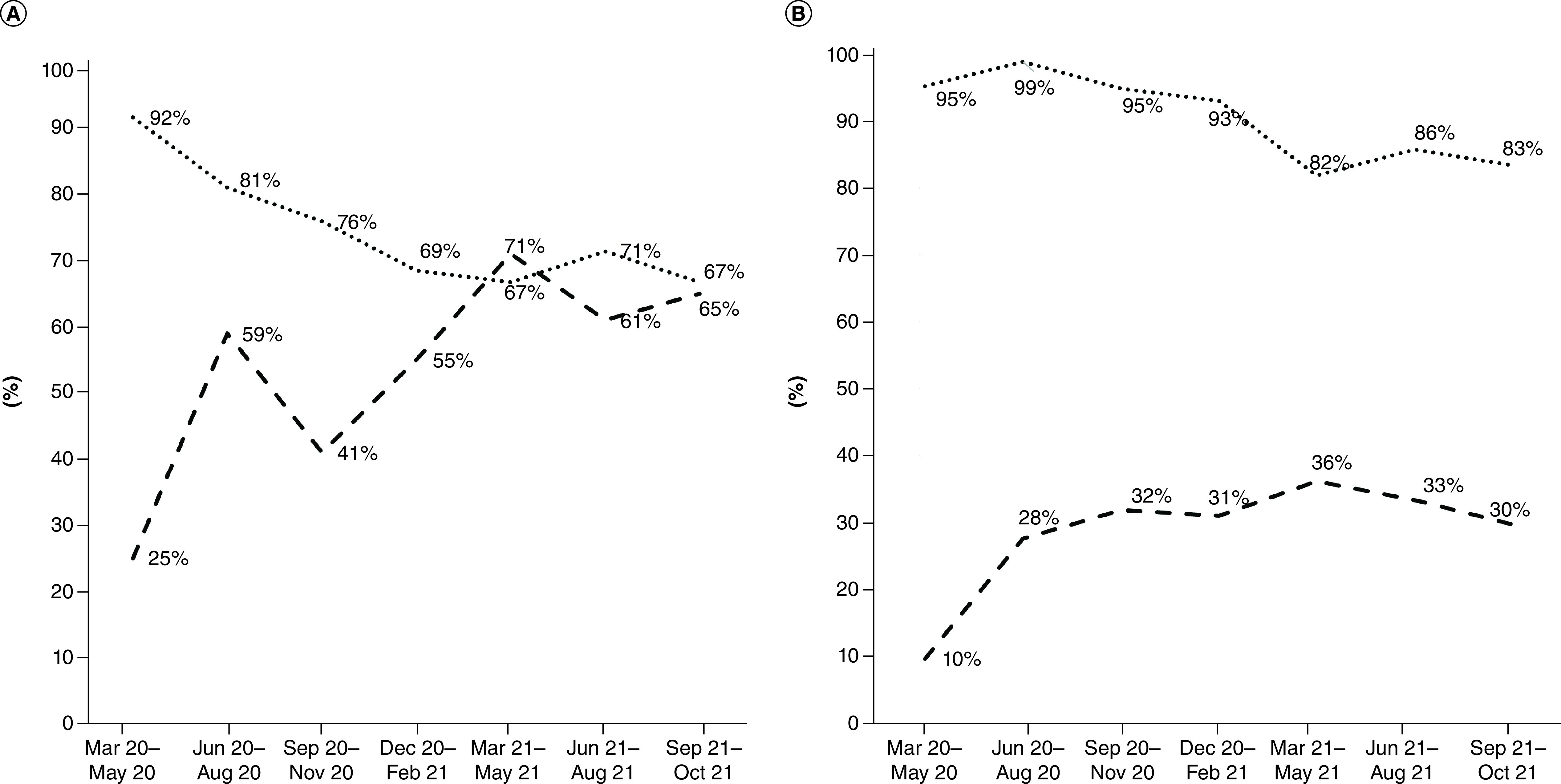
Receiver operator characteristic curves for lactate dehydrogenase cutoff values of 340 and 243 UI/l. **(A)** LDH 340 IU/l cutoff value; **(B)** LDH 243 IU/l cutoff value. LDH: Lactate dehydrogenase.

**Figure 4.**
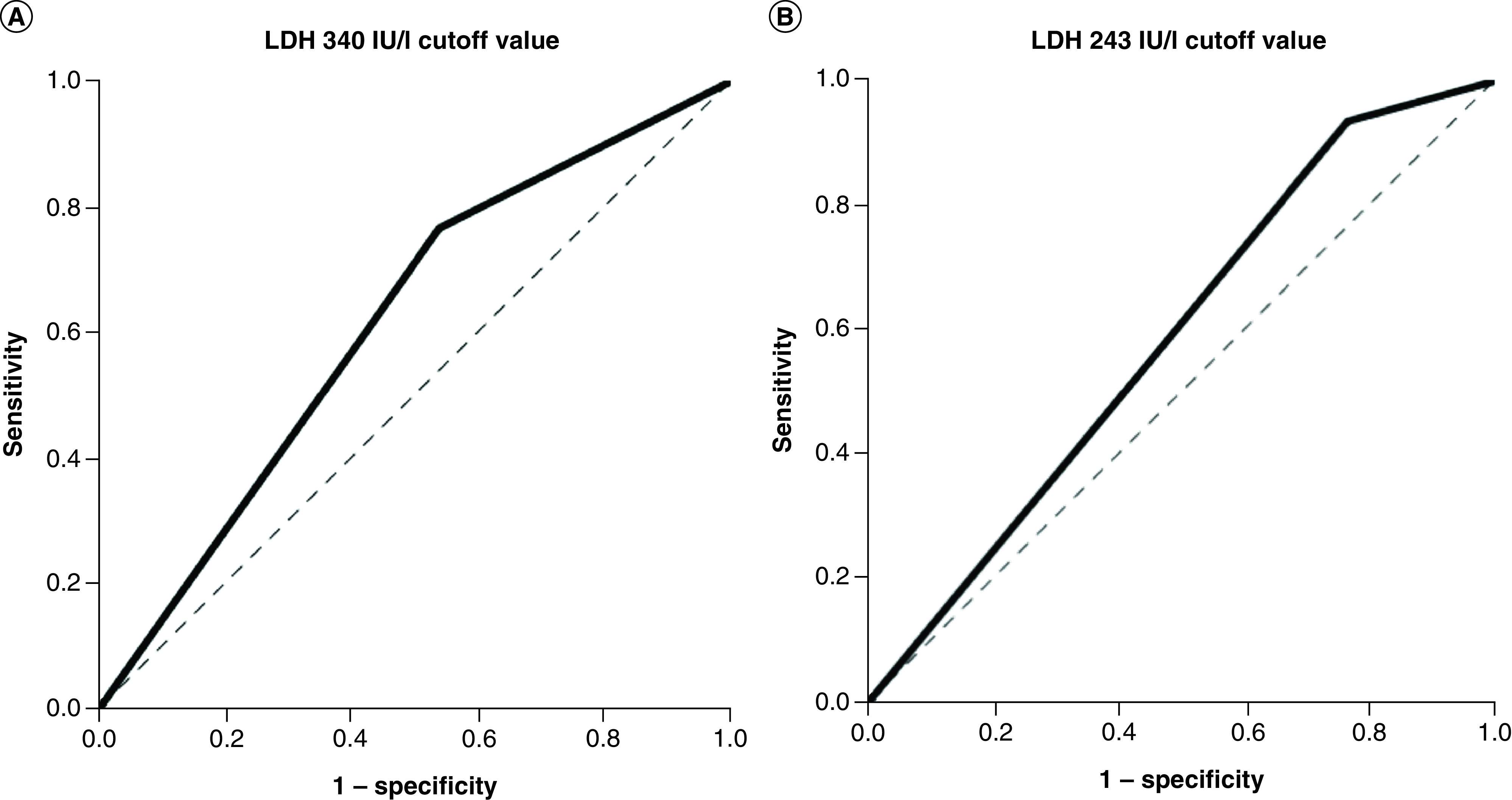
Sensitivity and specificity for LDH cutoff values of 340 and 243 IU/l per trimester. **(A)** Sensitivity and specificity for LDH 340 IU/l cutoff value per trimester. **(B)** Sensitivity and specificity for LDH 243 IU/l cutoff value per trimester. LDH: Lactate dehydrogenase.

The cutoff value for the second trimester (340 IU/l) was selected because it was similar to those described in previous reports [26–29]. For comparison and corroboration purposes, the value of the seventh trimester (243 IU/l) was also selected, similar to others previously reported [30]. Once these cutoff values were selected, ROC curves (Figure 3) and sensitivity/specificity were calculated trimester by trimester (Figure 4).

Finally, the RR for mortality was calculated for both cutoff values by trimester (Table 4).

**Table 4.** Relative risk for mortality by trimester.

Trimester	340 IU/l			243 IU/l		
	RR	95% CI	p-value	RR	95% CI	p-value
Total n = 843	2.80	2.09–3.77	<0.001	4.42	2.84–6.87	<0.001
Mar 20–May 20, n = 228	3.58	1.51–8.48	0.004	2.11	0.67–6.63	0.3
Jun 20–Aug 20, n = 113	6.02	2.40–15.07	<0.001	31.61	3.74–266.9	0.001
Sep 20–Nov 20, n = 138	2.17	0.84–5.62	0.109	8.55	2.53–27.83	0.001
Dec 20–Feb 21, n = 188	2.63	1.41–4.89	0.002	5.80	2.45–13.72	<0.001
Mar 21–May 21, n = 80	4.71	1.81–12.26	0.001	2.55	0.87–7.40	0.13
Jun 21–Aug 21, n = 47	3.84	0.994–14.88	0.051	3	0.56–15.82	0.28
Sep 21–Oct 21, n = 49	3.69	0.932–14.632	0.063	2.11	0.39–11.2	0.47

RR: Relative risk.

## Discussion

### Epidemiologic behavior of the studied patients

In the first stage of the pandemic (trimesters 1–4 of the current study), a markedly higher proportion of male patients were infected, according to previous reports [31,32]. However, this difference disappeared beginning in trimester 5 of the current study. As the pandemic evolved, this difference in infection susceptibility due to sexual dimorphism became less relevant than data indicating that men were more likely to have complications or die [33], therefore, beginning in trimester 5, sex ceased to be a protective factor for infection, but not for complications/death. Concerning age, the population in this study was younger than that in other studies [26,34]. This could be explained by the high prevalence of comorbidities in the Mexican population (e.g., type 2 diabetes, hypertension and obesity) [35]. Nevertheless, in the last two trimesters, the mean age decreased and showed no significant changes. This could indicate that, as time passed, the risk of infection did not necessarily relate to older age, but perhaps to the variability of COVID-19 itself, which is discussed later.

### LDH & pandemic waves

The fourth trimester of this study (December 2020–February 2021) was concurrent with the second pandemic wave, as noted by the Mexican Secretariat of Health (week 47, 2020 to week 8, 2021) [36]. Contrastingly, in this study, LDH levels peaked in the third trimester and showed a steep decline in the fourth trimester and this decrease continued until the last trimester of the study. This could have two possible explanations:

- Vaccinations began in Mexico on 24 December 2020. Vaccines exert a balancing effect between immune and adaptive responses, accelerating the latter. This could have elicited smoother immune responses that in turn caused more moderate immune imbalances. Given that such imbalances are responsible for the cytokine storm, a life-threatening general hyperinflammation state, this could explain the reduction observed in LDH in the last three trimesters compared with the first, and especially in the second wave;

- The first case of infection by the SARS-CoV2 Delta variant was reported in Mexico on 5 July 2021 [37]. It is feasible that LDH levels are affected in different manners depending on the viral variant implied. Unfortunately, viral typification in major public hospitals in Mexico is scarce; therefore, more studies are needed to confirm the possible connection between viral variants and blood LDH levels.

It is important to note that, at our hospital, the standard treatment for COVID-19 has not changed since the early days of the pandemic and consists of corticosteroids and anticoagulants. This is mainly because our institution is the largest government healthcare organization for the population not covered by social security. This particular situation limits regular access to newer and more effective treatments. Therefore, the changes observed in LDH levels are not attributable to therapeutic changes, but to the variables speculated upon earlier in this section.

### LDH decrease & mortality

As shown in Table 2 & Figure 2, we corroborated the assumption that LDH decreased during the pandemic. In accordance with previous reports [38,39], LDH was higher in nonsurvivors. However, it remains unclear if this finding was related to changes in survival rates, and if so, how this impacted the performance of LDH as an indicator capable of predicting mortality.

### Prognostic value of LDH for mortality

At the lower cutoff value for LDH (243 IU/l), most studied patients (725/843, 86%, p < 0.001) had elevated levels, whereas for the higher cutoff point (560/843, 66.4%, p < 0.001), the number of cases substantially decreased. The trimester-by-trimester RR analysis (Table 4) showed that the 340 IU/l cutoff value for LDH was not significant for trimesters 3, 6 and 7, whereas sensitivity/specificity analysis indicated a substantial loss of sensitivity (Figure 4). On the other hand, despite the sensitivity/specificity analysis for the 240 IU/l cutoff value showing acceptable percentages to be considered a predictor of mortality, the RR values for trimesters 3, 5, 6 and 7 were not statistically significant, nor was CI validity. Furthermore, neither of the two selected cutoffs had an AUC that could reliably discriminate between patients [40], unlike in other studies, such as that conducted by Han *et al.* [41], who reported very high sensitivity (96.9%) at a cutoff of 344.5 IU/l with an AUC of 0.89. Moreover, the CIs for the LDH means by trimester for survivors and nonsurvivors overlap in the fifth, sixth and seventh trimesters, suggesting it is an unreliable predictor of mortality. Finally, the Youden index for both values was not only considerably low from the first trimester (>0.5) but continued to decrease across trimesters, to 0.13 in the last trimester, confirming that, in the studied patients, the observed decrease in blood LDH substantially affected its mortality prediction capability.

### Sample size considerations & limitations

Despite sample sizes varying substantially between trimesters (especially trimesters 5, 6 and 7), they reflect the progressive decline in confirmed cases observed beginning in September 2021 and continuing until the second week of December 2021, prior to the fourth pandemic wave (which occurred after this study). Consequently, an FDR test was used and a power assessment of all trimesters was performed *a posteriori*, resulting in values ≥0.93, with the exception of the last trimester, which had a power of 0.74 (data not shown). This is likely explained by the fact that, due to the origin of the data, this period comprised only 2 months instead of 3. The application of the selection criteria could also have caused a bias and comorbidities were not considered. Finally, given that this was a retrospective study, the findings should be taken as preliminary, and more validation studies are needed.

## Conclusion

For reasons that remain to be explained, blood LDH levels upon admission for COVID-19 patients decreased throughout the study period. Moreover, this decrease provoked a loss of the predictive capacity of LDH for mortality. This finding could cast doubt on the usefulness of LDH as a reliable element in the use and development of mortality prediction scores, which may be necessary for critical resource assignation purposes, should new COVID-19 variants appear and outbreaks occur. Indeed, more studies are needed to support these preliminary findings.

**Summary points**

- Blood lactate dehydrogenase (LDH) levels in COVID-19 patients upon admission decreased over the first 20 months of the pandemic.
- This decrease has provoked a loss of the predictive capacity of LDH for mortality.
- The usefulness of LDH as a reliable element in the use and development of mortality prediction scores should be questioned.
- Mortality prediction scores are critical for resource assignation in COVID-19 pandemic peaks or outbreaks, especially in countries with scarce resources.
- LDH may no longer be a reliable marker for building mortality predicting scores, causing failures in the assignation of critical resources.
